# HbA1c as a Key Metabolic Marker in Predicting Myomectomy Requirement in Women with Uterine Fibroids: A Machine Learning Study

**DOI:** 10.3390/medicina62030500

**Published:** 2026-03-09

**Authors:** Inci Öz, Ecem E. Yegin, Ali Utku Öz, Engin Ulukaya

**Affiliations:** 1 Department of Gynaecology of Obstetrics, Medicana Atakoy Hospital, 34158 Istanbul, Türkiye; 2 Molecular Cancer Research Center, Istinye University, 34485 Istanbul, Türkiyeeulukaya@istinye.edu.tr (E.U.); 3 Department of Biostatistics and Medical Informatics, Faculty of Medicine, Istinye University, 34010 Istanbul, Türkiye; 4 Department of Gynaecology of Obstetrics, Cam & Sakura City Hospital, 34480 Istanbul, Türkiye; utkuoz@yahoo.com; 5 Department of Biochemistry, Faculty of Medicine, Istinye University, 34010 Istanbul, Türkiye

**Keywords:** uterine fibroids, timing of surgery, artificial intelligence, machine learning, HbA1c

## Abstract

*Background and Objectives*: Uterine fibroids are common benign tumors that frequently require surgical management, particularly myomectomy, in women of reproductive age. Metabolic dysfunction and insulin resistance have been implicated in fibroid biology; however, the clinical relevance of glycated hemoglobin (HbA1c) in predicting myomectomy requirement remains unclear. This study aimed to evaluate the predictive role of HbA1c for myomectomy requirement in women with uterine fibroids using conventional statistical analyses and machine learning-based models under real-world clinical decision-making conditions. *Materials and Methods*: This study evaluated data from a retrospective multicenter cohort comprising 618 women with a diagnosis of uterine fibroids. Patients were stratified according to myomectomy status (performed vs. not performed). Comparative analyses, univariate and multivariate logistic regression, and machine learning modeling were conducted using demographic, laboratory, hormonal, and fibroid-related variables. A total of 155 machine learning models were trained, and the top 20 models with the highest accuracy were evaluated. Blinded concordance analysis was conducted on 50 independent, anonymized cases evaluated by a gynecologist who was blinded to the study data. *Results*: Patients undergoing myomectomy (38.5%) had significantly higher HbA1c levels than non-surgical patients (5.57 ± 0.32 vs. 5.03 ± 0.61, *p* < 0.001). HbA1c showed a strong association with myomectomy requirement in univariate analysis (OR 0.026, 95% CI 0.012–0.055) but lost significance in multivariate models, while ferritin remained independently associated. Machine learning models incorporating HbA1c, ferritin, hormonal, and fibroid parameters achieved accuracies between 0.99 and 1.00. Blinded concordance analysis demonstrated 94% concordance between model predictions and expert clinical judgment. *Conclusions*: HbA1c is a valuable integrative marker in predicting myomectomy requirement when evaluated within multidimensional machine learning frameworks, although its independent effect is confounded by iron-related parameters. These findings support the use of HbA1c as part of a comprehensive decision-support approach in uterine fibroid management.

## 1. Introduction

Uterine fibroids, or uterine leiomyomas, are the most prevalent benign tumors affecting the female reproductive tract and constitute a significant source of gynecologic morbidity globally. They affect up to 70–80% of women during their reproductive years and are a leading indication for myomectomy and hysterectomy, particularly among women with fertility concerns or severe symptom burden [1,2]. Although fibroids are histologically benign, their clinical impact is substantial due to abnormal uterine bleeding, anemia, pelvic pain, bulk-related symptoms, and impaired reproductive outcomes.

The pathogenesis of uterine fibroids is complex and multifactorial, involving hormonal, genetic, and metabolic mechanisms. Estrogen and progesterone play central roles in fibroid growth by promoting cellular proliferation, extracellular matrix deposition, and local paracrine signaling within myometrial tissue [1,3]. Beyond classical sex steroid effects, growth factors such as insulin-like growth factor-I (IGF-I) and downstream signaling pathways have been implicated as important modulators of leiomyoma development and maintenance [2,4].

Increasing evidence suggests that metabolic dysfunction may contribute to uterine fibroid biology. Epidemiologic and case–control studies have demonstrated associations between uterine fibroids and components of the metabolic syndrome, including obesity, hypertension, dyslipidemia, and hyperglycemia [5,6,7]. These findings support the hypothesis that fibroids may share pathogenic pathways with cardiometabolic disease, potentially mediated through insulin resistance, chronic inflammation, and altered sex hormone bioavailability.

The relationship between insulin resistance, diabetes, and uterine fibroids, however, remains paradoxical. Experimental and animal studies have shown that insulin resistance can amplify estrogen and progesterone-driven myometrial growth by increasing expression of estrogen and progesterone receptors and proliferative markers [8]. In contrast, population-based studies have reported inverse or neutral associations between overt diabetes and fibroid prevalence, particularly among women treated with metformin [4,9]. These seemingly conflicting observations suggest that insulin resistance and its treatment may exert differential effects on fibroid initiation versus progression.

Metformin, the most widely used insulin-sensitizing agent, has emerged as a potential modifier of fibroid biology. Experimental studies have demonstrated that metformin directly inhibits leiomyoma cell proliferation through activation of AMP-activated protein kinase (AMPK) and suppression of the mTOR signaling pathway [10]. Consistent with these mechanistic findings, large population-based cohort studies have shown a significantly lower risk of uterine leiomyoma among women with type 2 diabetes who use metformin compared with non-users, suggesting a protective role of metabolic modulation in fibroid development [11].

In addition to insulin resistance, iron metabolism and anemia may play important roles in fibroid-related clinical decision-making. Heavy menstrual bleeding associated with fibroids frequently leads to iron deficiency and anemia, which may in turn influence metabolic biomarkers such as glycated hemoglobin (HbA1c). Ferritin levels have been shown to correlate with insulin resistance in women, independently of body mass index and menopausal status, highlighting a sex-specific interaction between iron metabolism and glucose homeostasis [12]. Moreover, obesity and metabolic dysregulation are associated with alterations in gonadotropins and sex steroid hormones, including lower follicle-stimulating hormone (FSH) and luteinizing hormone levels, which may further modulate fibroid growth dynamics [13].

HbA1c is widely used as a marker of long-term glycemic status and insulin resistance; however, its interpretation may be confounded in women with uterine fibroids due to coexisting anemia and iron deficiency. This complex interplay raises important questions regarding whether HbA1c reflects true metabolic risk, fibroid-related anemia, or a combination of both in patients evaluated for surgical management.

In women of reproductive age, myomectomy carries important implications for fertility, pregnancy outcomes, and future uterine integrity. Therefore, accurate identification of patients who may benefit from surgical intervention is essential not only for symptom control but also for appropriate reproductive counseling and shared decision-making. Predictive approaches that support individualized surgical planning may help optimize counseling and management strategies in this population [14,15].

Therefore, the aim of this study was to evaluate the role of HbA1c as a predictive marker for myomectomy requirement in women with uterine fibroids by integrating conventional statistical analyses and machine learning-based models, while accounting for hormonal, hematologic, and fibroid-related characteristics under real-world clinical decision-making conditions.

## 2. Materials and Methods

### 2.1. Ethical Consideration

This study was conducted following approval from the Istinye University Ethics Committee (approval date: 30 June 2025; decision number: 24-18). All procedures complied with the ethical principles of the Declaration of Helsinki. Due to the retrospective design, informed consent was waived.

### 2.2. Patients

The study population consisted of 618 women diagnosed with uterine fibroids who presented to participating obstetrics and gynecology departments of Private Derindere Hospital, Ataköy Medicana Hospital, and Kızılay Kağıthane Hospital in Türkiye clinics between 2019 and 2024. Patients were included if complete demographic data, laboratory measurements, and fibroid-related characteristics were available. The cohort was stratified according to myomectomy status, with 238 patients (38.5%) undergoing myomectomy and 380 patients (61.5%) managed without surgical intervention.

HbA1c measurements were obtained as part of routine metabolic and preoperative laboratory evaluation in the participating centers rather than as a fibroid-specific diagnostic test. The measurement reflected long-term glycemic status rather than acute metabolic changes. No cycle-based timing restrictions were applied for laboratory assessments. Patients with missing HbA1c values or incomplete key variables required for regression or machine learning analyses were excluded.

### 2.3. Study Design

This was a national, multicenter, retrospective observational study designed to evaluate the role of HbA1c in predicting the need for myomectomy in patients with uterine fibroids. The analytical strategy consisted of descriptive cohort characterization, comparative analysis between myomectomy and non-myomectomy groups, regression-based statistical modeling, and machine learning-based predictive modeling.

Baseline demographic, hormonal, hematologic, and fibroid-related variables were summarized for the entire cohort. Comparative analyses between patients with and without myomectomy were performed to identify variables associated with surgical decision-making.

### 2.4. Outcome Definition

The primary outcome variable was myomectomy status (performed vs. not performed). This outcome was defined based on real-world clinical decisions documented in medical records and was used as a surrogate for surgical necessity rather than an objective biological indication. Surgical decision-making in uterine fibroid management is multifactorial and may reflect symptom severity, fibroid burden, fertility considerations, and physician judgment, which are not uniformly quantifiable in retrospective datasets.

### 2.5. Statistical Analyses

Data distribution was assessed using skewness, kurtosis, and the Shapiro–Wilk test. Continuous variable distributions were visually inspected using histograms and Q–Q plots in addition to formal normality testing. Parametric or non-parametric tests were applied as appropriate based on distributional characteristics. Continuous variables were expressed as mean ± standard deviation, while categorical variables were reported as counts and percentages. Group comparisons were performed using the independent samples *t*-test or Mann–Whitney U test for continuous variables and the Chi-square test or Fisher’s exact test for categorical variables, as appropriate.

Univariate logistic regression analyses were first conducted to evaluate the association between individual input variables and myomectomy status. Variables demonstrating statistical significance in univariate analysis were subsequently included in a multivariate logistic regression model to assess their independent effects while adjusting for potential confounders. HbA1c was entered into regression models as a continuous variable in its original measurement scale without centering, inversion, or standardization. Therefore, reported odds ratios reflect the estimated change in outcome odds per one-unit increase in HbA1c. Regression coefficients (β), odds ratios (ORs), 95% confidence intervals, and *p* values were reported. A two-sided *p* value < 0.05 was considered statistically significant. Because variables were selected based on clinical relevance and predefined hypotheses, formal multiple-comparison correction procedures were not applied. Results were interpreted cautiously in the context of exploratory analysis and multivariable modeling.

Missing data were minimal and were handled using complete-case analysis without imputation. No variable included in regression or machine learning modeling exceeded a predefined missingness threshold.

### 2.6. Machine Learning Procedure and Pipeline

**Figure 1:**
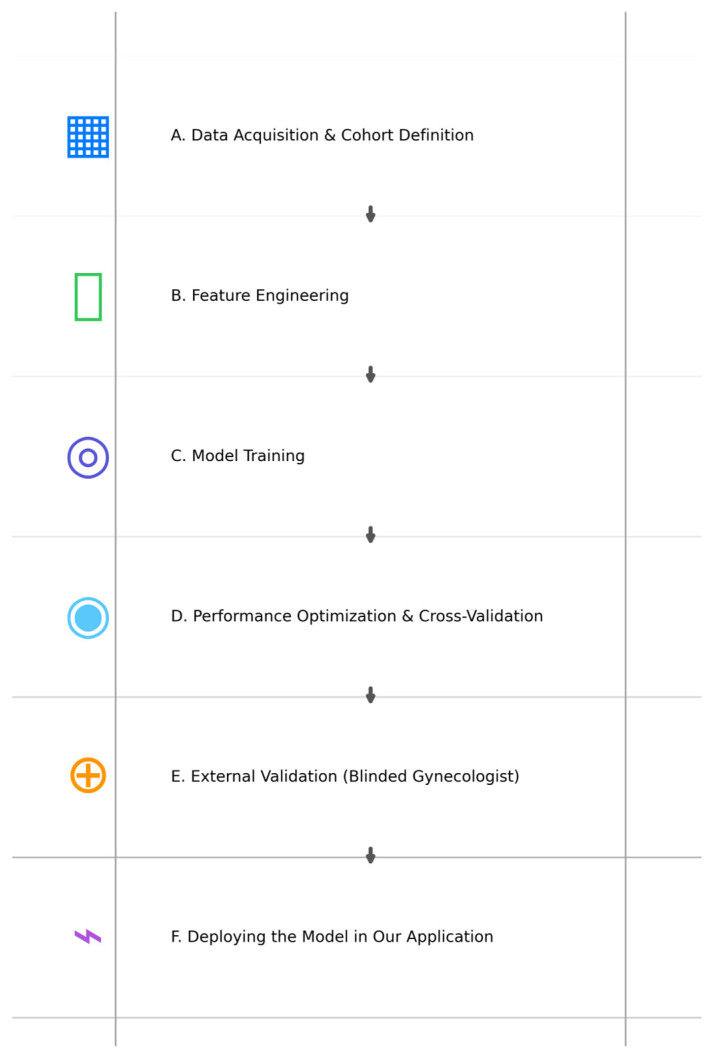
Machine learning-based clinical decision support workflow for predicting myomectomy requirements. The figure illustrates the sequential analytical pipeline used in this study. (**A**) Data acquisition and cohort definition from multicenter retrospective clinical records. (**B**) Feature engineering, including preprocessing and selection of demographic, metabolic, hormonal, hematologic, and fibroid-related variables. (**C**) Model training using multiple machine learning algorithms. (**D**) Performance optimization and internal validation through cross-validation and accuracy-based model selection. (**E**) Blinded concordance analysis using independent anonymized cases evaluated by a blinded gynecologist to assess clinical concordance. (**F**) Deployment of the final optimized model within a clinical application to support real-world decision-making.

Following completion of conventional statistical analyses, machine learning modeling was performed to evaluate multidimensional predictive patterns. Myomectomy status served as the target variable. A total of 155 machine learning models were trained using different combinations of clinical and laboratory input variables, including HbA1c, ferritin, age, hormonal parameters, and fibroid characteristics. Machine learning procedure and pipeline presented in Figure 1.

Model training and evaluation were conducted using a stratified train–test split with cross-validation performed within the training data only. The independent test set and blinded concordance analysis cases were not used during model training or model selection. Despite these precautions, the relatively structured nature of the dataset and strong separation of certain variables may produce optimistic performance estimates. Therefore, the machine learning analysis should be interpreted as exploratory rather than as a finalized predictive modeling framework.

No automated feature selection procedure was performed prior to data splitting. Instead, combinations of clinically relevant variables were predefined and evaluated across multiple models. The dataset was first divided into training and test sets using stratified sampling, and all model training and cross-validation procedures were conducted exclusively within the training set. Therefore, no feature selection was performed on the full dataset before splitting, and the risk of information leakage from feature selection procedures was minimized.

Multiple supervised classification algorithms were employed, including logistic regression, support vector machine, decision tree, random forest, and k-nearest neighbors. A stratified 70:30 train–test split was applied to preserve class proportions. Five-fold cross-validation was performed within the training set to enhance model stability. Model performance was evaluated using accuracy, area under the receiver operating characteristic curve, precision, recall, and F1 score. Because the dataset demonstrated moderate class imbalance, model performance was assessed using multiple complementary metrics rather than relying on accuracy alone. The 20 highest-performing models, all demonstrating accuracy values greater than 99%, were selected for presentation.

Model calibration analyses, including Brier scores or reliability curves, were not conducted in this retrospective dataset. Because calibration assessment requires larger, prospective, and more uniformly documented cohorts, and because the machine learning component was designed as an exploratory decision-support analysis rather than as a finalized clinical prediction model intended for immediate clinical deployment, this component is planned for future prospective validation studies. The absence of calibration analysis is acknowledged as a methodological limitation of the present work.

### 2.7. Blinded Concordance Analysis Procedure

To assess concordance between model outputs and independent clinical judgment, an exploratory blinded concordance analysis was conducted using 50 anonymized clinical cases that were not included in the training dataset. These cases were independently reviewed by an experienced gynecologist who was blinded to both the model predictions and the actual surgical outcomes. The clinician was asked to determine whether myomectomy was indicated for each case based solely on the provided clinical information.

For consistency and interpretability, the validating gynecologist was provided exclusively with the same fibroid-related characteristics and laboratory parameters, including HbA1c, that were used as inputs for the machine learning model. No additional symptom-level data—such as abnormal uterine bleeding severity, pain, bulk-related symptoms, prior treatment response, or patient preferences—were disclosed during the validation process. This constrained information framework was intentionally applied to ensure that the concordance analysis reflected alignment between human clinical judgment and model predictions under comparable informational conditions. Because the concordance analysis involved a single blinded gynecologist rather than multiple independent raters, formal inter-rater reliability statistics such as Cohen’s kappa could not be calculated. This represents a methodological limitation of the present concordance analysis and should be addressed in future multi-rater validation studies.

Given that the blinded concordance analysis cohort was limited to 50 cases and that the validating clinician was provided only with the structured variables used as model inputs—without access to symptom severity, patient preferences, prior treatment history, or other contextual clinical information—this analysis should be interpreted as an exploratory concordance assessment rather than a statistically powered validation of real-world surgical decision-making. These constraints represent methodological limitations and should be considered when interpreting the findings. Larger, prospective, multi-center cohorts with comprehensive clinical information will be required to establish generalizability and clinical reliability.

### 2.8. Class Distribution and Handling of Imbalance

The dataset demonstrated moderate class imbalance between myomectomy and non-myomectomy groups. No synthetic oversampling, undersampling, or cost-sensitive weighting techniques were applied. This decision was made to preserve real-world clinical decision patterns and avoid artificial performance inflation. Stratified sampling and the use of multiple complementary performance metrics were employed to mitigate potential bias.

### 2.9. Hyperparameter Configuration

ML models were trained using default hyperparameters without additional tuning. This approach was chosen to prioritize clinical interpretability and to assess whether observed performance reflected intrinsic data structure rather than optimization-driven effects. Model stability was evaluated using cross-validation and blinded concordance analysis.

Because the primary aim was exploratory identification of clinically relevant parameter interactions rather than development of a deployment-ready predictive model, systematic hyperparameter tuning was intentionally not performed. While this approach reduces optimization-driven overfitting, it may limit robustness assessment and is acknowledged as a study limitation. Future studies using larger prospective datasets should incorporate systematic hyperparameter optimization and calibration analyses.

### 2.10. Software and Reporting Standards

All statistical analyses and machine learning procedures were conducted using Wistats v3.0 (WisdomEra Corp., Istanbul, Turkey), incorporating Python v2.7.14-based libraries including SciPy, scikit-learn, and statsmodels. The study was designed and reported in alignment with TRIPOD-AI [16] (Supplementary Table S1) and PROBAST-AI [17] methodological principles for transparent reporting and bias-aware evaluation of clinical prediction models.

### 2.11. Deploying the Decision Support Algorithm in a Web-Based Environment

**Figure 2:**
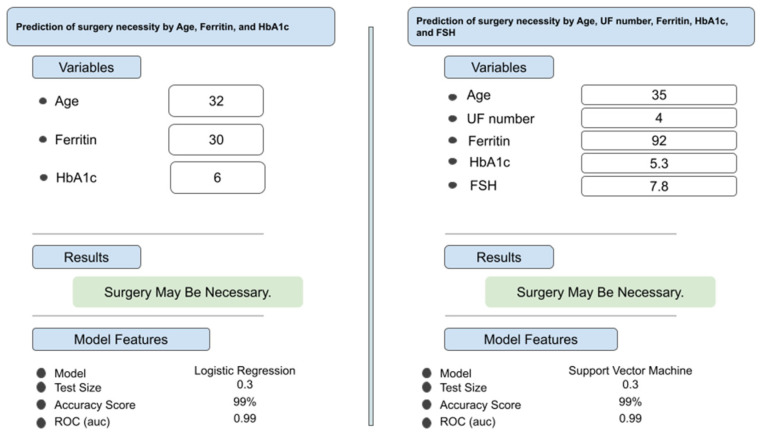
Representative clinical decision support interface for predicting myomectomy necessity using machine learning models. The figure demonstrates two example prediction scenarios generated by the developed decision support system. The left panel illustrates a model based on age, ferritin, and HbA1c, analyzed using logistic regression, while the right panel shows a more comprehensive model incorporating age, uterine fibroid number, ferritin, HbA1c, and FSH, analyzed using a support vector machine. For each scenario, patient-specific input variables are entered, and the system outputs a binary clinical recommendation (“Surgery may be necessary”). Model characteristics, including test set proportion, accuracy, and area under the receiver operating characteristic curve (ROC–AUC), are displayed to enhance transparency and interpretability. These examples highlight the real-world applicability of machine learning-based tools in supporting individualized surgical decision-making.

To enable independent reproducibility testing, the highest-performing model was made accessible through a web-based interface (available at JinekoAI.com), which functions solely as an input–output layer without modifying any internal model parameters. The model itself runs on the WisdomEra cloud-based analytics infrastructure (available at WisdomEra.io), which provides the standardized computational backend required for real-time inference. Communication between the interface and the trained model occurs via a secure API, ensuring that predictions are produced exactly as generated by the original machine learning pipeline (Figure 2). This setup allows the model to be evaluated by external users under controlled and reproducible technical conditions while avoiding any form of manual post-training adjustment.

The model used for analysis corresponds to a fixed research version that was frozen prior to validation and manuscript preparation. The web-based interface represents a research prototype, and version control was maintained within the development environment to ensure reproducibility of results. Future iterations will incorporate formal version tracking and deployment-level documentation.

## 3. Results

**Figure 3:**
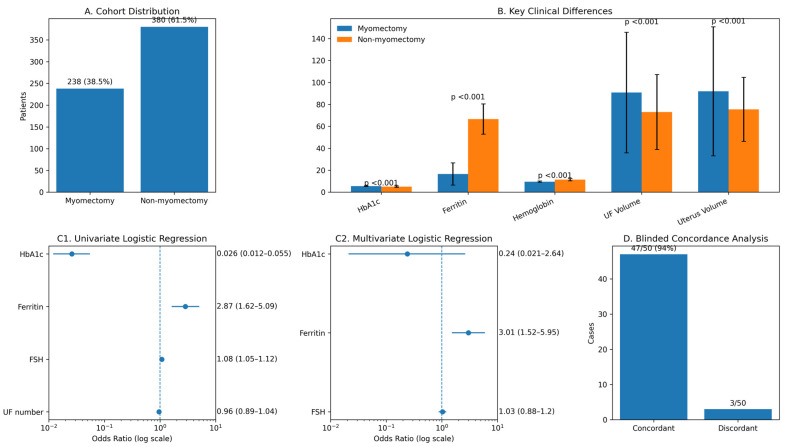
HbA1c-integrated prediction of myomectomy requirement in women with uterine fibroids. (**A**) Cohort distribution of the study population. Among 618 women with uterine fibroids, 238 patients (38.5%) underwent myomectomy, whereas 380 patients (61.5%) were managed without surgical intervention. (**B**) Key clinical, laboratory, and fibroid-related differences between myomectomy and non-myomectomy groups. Patients undergoing myomectomy exhibited significantly higher HbA1c levels and markedly lower hemoglobin and ferritin levels, accompanied by significantly larger uterine fibroid and uterine volumes. Bars represent mean values with standard deviation error bars; *p* values indicate between-group comparisons. (**C1**) Univariate logistic regression shows a strong association of HbA1c, ferritin, and follicle-stimulating hormone (FSH) with surgical decision-making. (**C2**) Multivariate logistic regression indicates that ferritin remains independently associated with myomectomy requirement after adjustment, whereas HbA1c and FSH lose statistical significance, suggesting confounding and interrelated metabolic–hematologic effects. Odds ratios are displayed on a logarithmic scale with 95% confidence intervals. (**D**) Blinded concordance analysis of the decision-support model. Comparison of model predictions with blinded expert clinical judgment in 50 independent cases demonstrated concordant myomectomy decisions in 47 cases (94%), supporting exploratory concordance between model outputs and expert clinical judgment under constrained real-world informational conditions.

A total of 618 patients with uterine fibroids were included in the analysis. Among them, myomectomy was performed in 238 patients (38.5%), while 380 patients (61.5%) were managed without surgical intervention (Figure 3A). The baseline demographic, laboratory, hormonal, and fibroid-related characteristics of the overall cohort are summarized in Table 1. The mean age of the cohort was 35.48 ± 7.06 years, and the mean HbA1c level was 5.24 ± 0.58. Fibroid burden was substantial, with a mean uterine fibroid number of 4.64 ± 2.17 and a mean fibroid volume of 79.89 ± 44.22 cm^3^.

### 3.1. Comparative Analysis Between Myomectomy and Non-Myomectomy Groups

Comparative analyses revealed significant differences between patients who underwent myomectomy and those who did not (Table 2). HbA1c levels were significantly higher in the myomectomy group compared with the non-myomectomy group (5.57 ± 0.32 vs. 5.03 ± 0.61, *p* < 0.001). Markers related to anemia and iron status also differed markedly; ferritin and hemoglobin levels were significantly lower in patients who underwent myomectomy (both *p* < 0.001). Hormonal parameters, including FSH, LH, E2, prolactin, and AMH, demonstrated statistically significant between-group differences, with lower FSH and higher AMH levels observed in the myomectomy group (Figure 3B). Fibroid-related characteristics showed that patients undergoing myomectomy had significantly larger uterine fibroid volume and uterine volume (both *p* < 0.001), whereas differences in disease duration and gravidity were not statistically significant. Pregnancy desire and parity differed significantly between groups, with a higher proportion of pregnancy desire among patients who underwent myomectomy.

### 3.2. Univariate and Multivariate Logistic Regression Analyses

In univariate logistic regression analysis, HbA1c was significantly associated with myomectomy requirement (OR 0.026, 95% CI 0.012–0.055, *p* < 0.001) (Table 3). Ferritin (OR 2.87, *p* < 0.001) and FSH (OR 1.08, *p* < 0.001) were also significantly associated with myomectomy status, whereas uterine fibroid number was not significant (Figure 3C1,C2). In the multivariate logistic regression model including significant univariate predictors, ferritin remained independently associated with myomectomy requirement (OR 3.01, 95% CI 1.52–5.95, *p* = 0.002). In contrast, HbA1c and FSH lost statistical significance after adjustment.

The large magnitude of the univariate odds ratio for HbA1c reflects the estimated effect per one-unit increase in HbA1c entered as a continuous variable in its original scale. Given the relatively narrow physiological range of HbA1c values in this cohort, a one-unit increment represents a substantial metabolic shift, which may produce numerically extreme odds ratios in unadjusted models. The disappearance of this association in multivariate analysis suggests that the univariate estimate is influenced by interrelated metabolic and hematologic parameters rather than representing an independent causal effect.

**Table 3:** Univariate and Multivariate Analyses of Input Variables for Predicting Myomectomy.

	Variable	β	OR	%95 CI	*p*	Effect
Univariate	UF number	−0.032	0.96	0.89–1.04	0.390	Not significant
	HbA1c	−3.635	0.026	0.012–0.055	<0.001	**Strong**
	FSH	0.079	1.08	1.05–1.12	<0.001	Modest
	Ferritin	1.054	2.87	1.62–5.09	<0.001	**Strong**
Multivariate	FSH	0.029	1.03	0.88–1.20	0.696	Not significant
	Ferritin	1.102	3.01	1.52–5.95	0.002	**Strong**
	HbA1c	−1.426	0.24	0.021–2.64	0.244	Not significant

### 3.3. Machine Learning Model Performance

A total of 155 machine learning models were trained using different combinations of clinical, laboratory, and fibroid-related variables. The top 20 models, all achieving accuracy values greater than 99%, are presented in Table 4. Across these models, combinations including ferritin, age, uterine fibroid number, FSH, and HbA1c consistently demonstrated excellent predictive performance. Accuracy ranged from 0.99 to 1.00, with corresponding high AUC, precision, recall, and F1 scores, indicating consistently high classification performance across multiple algorithmic approaches within this dataset.

### 3.4. Blinded Concordance Analysis

Blinded concordance analysis was performed using 50 independent and anonymized clinical cases that were not included in the training dataset. When model predictions were compared with the blinded clinical judgment of an experienced gynecologist, concordant decisions regarding myomectomy indication were observed in 47 of 50 cases (94%) (Figure 3D). This high level of agreement supports the external decision-support capability of the developed model under real-world informational constraints and suggests strong alignment between model-based predictions and expert clinical assessment.

## 4. Discussion

The present study aimed to evaluate the role of HbA1c as a predictive marker for myomectomy requirement in women with uterine fibroids by integrating conventional statistical analyses with machine learning-based modeling under real-world clinical decision-making conditions. In a cohort of 618 patients, myomectomy was performed in 38.5% of cases, and patients undergoing surgery exhibited significantly higher HbA1c levels than those managed conservatively (5.57 ± 0.32 vs. 5.03 ± 0.61, *p* < 0.001). In addition, surgically treated patients demonstrated lower ferritin and hemoglobin levels, altered sex hormone profiles, and substantially greater fibroid and uterine volumes. While HbA1c showed an association with myomectomy requirement in univariate regression (OR 0.026, 95% CI 0.012–0.055), this association lost significance in multivariate analysis, whereas ferritin remained an independent predictor. Machine learning models, however, consistently identified HbA1c among the most informative variables, achieving accuracy values exceeding 99%, and blinded concordance analysis demonstrated 94% concordance with expert clinical judgment.

In this study, the primary outcome reflects a physician-driven surgical decision rather than an objectively standardized biological threshold. Accordingly, the developed models should be interpreted as representations of real-world clinical decision behavior rather than direct measures of intrinsic fibroid severity. Surgical indication in uterine fibroid management arises from a multidimensional interaction among metabolic status, anemia, fibroid characteristics, fertility considerations, and clinician judgment. Therefore, the machine learning models are not intended to predict a single isolated construct such as biological severity, physician preference, or institutional practice patterns alone. Instead, the predicted outcome represents a composite clinical decision context reflecting patterns associated with surgical recommendation under routine care conditions. Modeling this composite pathway allows identification of factors linked to surgical decision-making without implying that the algorithm determines absolute disease severity or replaces individualized clinical assessment. This conceptual framing is important when interpreting model performance and underscores the role of machine learning tools as decision-support instruments rather than diagnostic arbiters.

The observed association between metabolic parameters and fibroid-related surgical decision-making is consistent with accumulating evidence that uterine fibroids share pathogenic pathways with metabolic dysfunction. Previous epidemiologic studies have demonstrated links between fibroids and components of the metabolic syndrome, including obesity, hypertension, dyslipidemia, and hyperglycemia, supporting the concept that fibroids may reflect a broader cardiometabolic phenotype [5,6,7]. Large cohort data from the SWAN study further suggested that insulin resistance may increase fibroid risk during premenopause, while diabetes—particularly when treated with metformin—may be associated with a lower incidence of fibroid diagnosis, highlighting a complex and stage-dependent metabolic influence [9]. In this context, the progression from molecular signaling to clinical decision-making can be understood as the cumulative effect of sustained metabolic and hormonal stress on uterine tissue. Persistent activation of proliferative pathways leads not only to fibroid enlargement but also to increased vascularization, extracellular matrix deposition, and tissue rigidity, all of which contribute to symptom severity and reduced responsiveness to conservative management. As fibroids grow under the influence of insulin-mediated and estrogen-dominant signaling, patients are more likely to experience heavy menstrual bleeding, pressure-related symptoms, and anemia, which further complicate metabolic homeostasis. This self-reinforcing cycle—where metabolic dysfunction accelerates fibroid growth and fibroid-related bleeding exacerbates systemic metabolic strain—progressively narrows non-surgical treatment options. Consequently, surgical intervention becomes a more frequent and clinically justified outcome, not solely due to fibroid size but as a result of intertwined metabolic, endocrine, and hematologic disturbances. Framing fibroid-related surgery within this mechanistic continuum highlights the importance of integrating metabolic assessment into preoperative risk stratification and supports the rationale for multidimensional decision-support models that capture these complex biological interactions.

Experimental data provide biological plausibility for these observations. Animal models have shown that insulin resistance can amplify estrogen- and progesterone-driven myometrial growth by increasing expression of estrogen and progesterone receptors and proliferative markers [8]. At the cellular level, metformin has been shown to inhibit leiomyoma cell proliferation via activation of AMP-activated protein kinase and suppression of mTOR signaling, suggesting a direct anti-fibroid effect independent of glycemic control [10]. These mechanistic findings are supported by population-based data indicating a substantially reduced risk of uterine leiomyoma among women with type 2 diabetes who use metformin compared with non-users (hazard ratio 0.47), reinforcing the relevance of insulin signaling pathways in fibroid biology [11]. Beyond these effects, insulin-mediated signaling has been shown to interact with local growth factor networks, enhancing extracellular matrix deposition and fibrotic remodeling within leiomyomas. Chronic hyperinsulinemia may also alter local steroid metabolism within uterine tissue, thereby sustaining a pro-proliferative hormonal microenvironment. In parallel, suppression of mTOR activity by metformin has been associated with reduced angiogenic signaling, potentially limiting fibroid vascularization and growth potential. These combined actions suggest that metabolic modulation can influence both cellular proliferation and the structural characteristics of fibroids. Collectively, these mechanisms provide a coherent biological framework linking insulin signaling to fibroid progression and therapeutic response.

In the present study, HbA1c emerged as a significant discriminator between myomectomy and non-myomectomy groups in univariate analysis and machine learning models yet lost independent significance in multivariate regression. This apparent discrepancy likely reflects the complex interaction between HbA1c, anemia, and iron metabolism in fibroid patients. Heavy menstrual bleeding associated with fibroids frequently leads to iron deficiency and anemia, conditions known to influence HbA1c measurements independent of true glycemic status. Multiple studies have demonstrated that iron deficiency anemia can distort HbA1c values, often resulting in falsely elevated or altered readings depending on anemia severity and measurement methodology [18,19,20]. Meta-analyses further confirm that iron replacement therapy can significantly reduce HbA1c levels, underscoring the need for cautious interpretation of HbA1c in patients with iron deficiency [21,22].

The independent association of ferritin with myomectomy requirement in multivariate analysis (OR 3.01, *p* = 0.002) highlights the importance of iron metabolism in this clinical context. Ferritin has been consistently linked to insulin resistance and metabolic syndrome in women, with sex-specific effects that persist after adjustment for body mass index and menopausal status [23,24]. In reproductive-age women and those with polycystic ovary syndrome, elevated ferritin levels have been shown to reflect insulin resistance and hyperinsulinemia rather than reduced menstrual blood loss, suggesting a bidirectional relationship between iron metabolism and metabolic dysfunction [25,26].

Alterations in sex hormone profiles observed in the myomectomy group, including lower FSH and higher AMH levels, are also consistent with known interactions between metabolic status and reproductive endocrinology. Obesity and insulin resistance have been shown to suppress gonadotropin secretion and alter estrogen dynamics, even in otherwise fertile women [13]. Moreover, accumulating evidence suggests that gonadotropins, prolactin, and sex steroids interact with glucose metabolism and insulin sensitivity, potentially modulating fibroid growth indirectly through endocrine–metabolic crosstalk [27,28,29].

The observed performance of the machine learning models in this study, with accuracies ranging from 0.99 to 1.00 and 94% concordance with blinded expert assessment, suggests that HbA1c retains clinically relevant information when interpreted in combination with other variables. Unlike traditional regression models, which aim to isolate independent effects, machine learning approaches can capture nonlinear interactions and joint patterns among metabolic, hormonal, and fibroid-related parameters. In this context, HbA1c may function as an integrative surrogate marker reflecting cumulative metabolic stress, anemia-related distortion, and endocrine alterations rather than a direct causal driver of fibroid growth. HbA1c values may be influenced by iron deficiency and should therefore be interpreted within the broader hematologic context, particularly in cohorts with significant differences in ferritin levels. Although iron deficiency is known to affect HbA1c measurements, stratified or corrected analyses accounting for iron status were not performed in this study. Therefore, HbA1c findings should be interpreted cautiously, and future investigations should incorporate iron-adjusted or stratified analyses to better delineate independent metabolic effects. The inverse association observed in univariate models should therefore not be interpreted as indicating a protective or causal metabolic effect but rather as reflecting the complex interaction between glycemic markers, iron metabolism, and clinical decision context. A recent nationwide population-based cohort study in young women also reported an association between uterine leiomyoma and incident type 2 diabetes mellitus, while women who underwent myomectomy did not demonstrate the same elevated diabetes risk, suggesting potential bidirectional interactions between fibroid burden and metabolic regulation [30].

Several limitations should be acknowledged. The retrospective design precludes causal inference and limits control over unmeasured confounders, including symptom severity, bleeding volume, and prior medical treatments. HbA1c measurements were not corrected for anemia severity or iron supplementation status, which may have influenced observed associations. Model calibration analyses were not performed, and the blinded concordance analysis involved a single blinded gynecologist, precluding inter-rater reliability assessment. A key limitation of this study is the absence of standardized criteria for myomectomy indication across participating clinical settings. Surgical decisions in uterine fibroid management were made according to routine clinical judgment rather than a uniform protocol, reflecting real-world practice but introducing heterogeneity in outcome definition. Consequently, the modeled endpoint represents a composite decision influenced by symptom burden, anemia, fertility considerations, and clinician assessment rather than a strictly standardized surgical threshold. This variability limits direct interpretation of the models as predictors of absolute surgical necessity and should be considered when evaluating the findings. The high accuracy values observed in several machine learning models should be interpreted cautiously. In moderate-sized retrospective datasets with strong separation between key predictors and outcomes, apparent near-perfect classification performance may occur even in the absence of direct data leakage. Nevertheless, the possibility of optimistic performance estimates or overfitting cannot be excluded. These models are therefore best viewed as exploratory tools reflecting patterns within this dataset rather than as fully generalizable predictive instruments. Prospective validation in larger and more heterogeneous cohorts will be required before clinical deployment can be considered. In addition, hyperparameter tuning was not performed, as the machine learning component was intended for exploratory pattern detection rather than predictive optimization. Future prospective studies with larger datasets should incorporate systematic hyperparameter tuning and calibration analyses. In addition, sensitivity analyses stratified by anemia status were not performed; future studies using predefined anemia categories and iron-adjusted modeling strategies may help clarify the independent contribution of HbA1c in this clinical context. The racial and ethnic background of patients was not systematically recorded in the retrospective dataset. Given that uterine fibroid prevalence and metabolic disease burden vary across populations, generalizability to more ethnically diverse cohorts may be limited. Formal collinearity diagnostics such as variance inflation factor analysis were not performed and should be incorporated in future regression-based modeling studies. Finally, although the machine learning models demonstrated high classification performance within this dataset, the possibility of overfitting cannot be fully excluded and the findings should be interpreted as exploratory rather than as evidence of generalizable robustness.

Future studies should incorporate prospective designs with standardized assessment of anemia, iron status, insulin resistance indices, and fibroid symptom burden. Longitudinal evaluation of HbA1c dynamics before and after anemia correction or metabolic intervention may help disentangle biological effects from measurement bias. Additionally, multi-center prospective validation with calibration analyses and multiple independent clinical raters would further strengthen the clinical applicability of AI-based decision-support tools in fibroid management.

## 5. Conclusions

In conclusion, this study demonstrates that HbA1c is significantly associated with the myomectomy requirement in women with uterine fibroids at the univariate and machine learning levels but not as an independent predictor after adjustment for iron-related parameters. Ferritin emerged as an independent marker within this dataset, highlighting the potential relevance of iron metabolism and anemia in fibroid-related clinical decision-making. Machine learning models integrated HbA1c with hormonal and fibroid-related features, yielding high classification performance and concordance with expert judgment within this dataset. These findings suggest that HbA1c should be interpreted as part of a multidimensional metabolic–endocrine profile rather than as a standalone marker when evaluating surgical necessity in uterine fibroid patients.

The intended use of the proposed modeling framework is exploratory clinical decision-support in research settings rather than direct clinical deployment. The models are not intended for standalone clinical decision-making and require prospective multi-center validation before implementation. Given the observational design of the study, findings should be interpreted as associative rather than causal and require further prospective validation before clinical inference.

## Figures and Tables

**Table 1:** Baseline Demographic and Clinical Characteristics of the Study Population.

	Cohort Mean ± SD, N (%)
Myomectomy Prediction	
surgery applied	238 (38.5%)
surgery not applied	380 (61.5%)
Age	35.48 ± 7.06 n = 618
HbA1c	5.24 ± 0.58 n = 618
Ferritin	47.39 ± 27.44 n = 618
Hemoglobin	10.69 ± 1.28 n = 618
FSH	9.48 ± 7.98 n = 618
LH	6.89 ± 4.94 n = 618
E2	43.44 ± 27.94 n = 618
Prolactine	12.12 ± 5.59 n = 618
Amh	2.43 ± 1.53 n = 618
UF number	4.64 ± 2.17 n = 618
UF Volume	79.89 ± 44.22 n = 618
Uterus Volume	81.84 ± 43.77 n = 618
Disease Duration (years)	
1–5	342 (55.3%)
>5	276 (44.7%)
Pregnancy Desire	
no	234 (37.9%)
yes	384 (62.1%)
Gravidity	
no	79 (12.8%)
yes	539 (87.2%)
Parity	
no	203 (32.8%)
yes	415 (67.2%)

n: number of cases.

**Table 2:** Case Characteristics and Comparative Results Between Patients With and Without Myomectomy.

	Surgery Applied Mean ± SD, N (%)	Surgery not Applied Mean ± SD, N (%)	*p*
Age	35.66 ± 5.34 n = 238	35.37 ± 7.96 n = 380	0.371
HbA1c	5.57 ± 0.32 n = 238	5.03 ± 0.61 n = 380	<0.001
Ferritin	16.55 ± 10.10 n = 238	66.71 ± 13.81 n = 380	<0.001
Hemoglobin	9.59 ± 0.53 n = 238	11.39 ± 1.10 n = 380	<0.001
FSH	7.29 ± 3.79 n = 238	10.86 ± 9.47 n = 380	<0.001
LH	6.07 ± 3.70 n = 238	7.41 ± 5.52 n = 380	0.008
E2	46.99 ± 9.04 n = 238	41.21 ± 34.74 n = 380	<0.001
Prolactine	10.56 ± 5.09 n = 238	13.10 ± 5.67 n = 380	<0.001
Amh	2.61 ± 1.44 n = 238	2.31 ± 1.57 n = 380	0.002
UF number	4.74 ± 1.76 n = 238	4.58 ± 2.39 n = 380	0.015
UF Volume	90.81 ± 54.95 n = 238	73.06 ± 34.24 n = 380	<0.001
Uterus Volume	91.93 ± 58.79 n = 238	75.52 ± 29.22 n = 380	<0.001
Disease Duration (years)			0.193
1–5	126 (52.9%)	216 (56.8%)	
>5	112 (47.1%)	164 (43.2%)	
Pregnancy Desire			<0.001
no	62 (26.1%)	172 (45.3%)	
yes	176 (73.9%)	208 (54.7%)	
Gravidity			0.166
no	26 (10.9%)	53 (13.9%)	
yes	212 (89.1%)	327 (86.1%)	
Parity			0.023
no	90 (37.8%)	113 (29.7%)	
yes	148 (62.2%)	267 (70.3%)	

n: number of cases.

**Table 4:** Top 20 Machine Learning Models With An Accuracy Score Greater Than 99%.

Input	Model	Accuracy	Roc	Precision	Recall	F Score
Age Ferritin	LR	0.99	1	1.0	0.97	0.99
Age Ferritin	SVM	0.99	0.99	1.0	0.97	0.99
Age Ferritin	DT	0.99	0.99	1.0	0.97	0.99
Age Ferritin	RF	0.99	0.99	1.0	0.97	0.99
Age Ferritin	KNN	0.99	0.99	1.0	0.97	0.99
Ferritin FSH	DT	0.99	0.99	1.0	0.97	0.99
Ferritin FSH	KNN	0.99	0.99	1.0	0.97	0.99
Age UF number Ferritin	SVM	0.99	0.99	1.0	0.97	0.99
Age UF number Ferritin	DT	0.99	0.99	1.0	0.97	0.99
Age UF number Ferritin	RF	0.99	0.99	1.0	0.97	0.99
Age UF number Ferritin	KNN	0.99	0.99	1.0	0.97	0.99
Age Ferritin HbA1c	LR	0.99	1	1.0	0.97	0.99
Age Ferritin HbA1c	SVM	0.99	0.99	1.0	0.97	0.99
Age Ferritin HbA1c	DT	0.99	0.99	1.0	0.97	0.99
Age Ferritin HbA1c	RF	0.99	0.99	1.0	0.97	0.99
Age Ferritin HbA1c	KNN	0.99	0.99	1.0	0.97	0.99
Age Ferritin FSH	DT	0.99	0.99	1.0	0.97	0.99
Age Ferritin FSH	RF	0.99	0.99	1.0	0.97	0.99
Age Ferritin FSH	KNN	0.99	0.99	1.0	0.97	0.99
UF number Ferritin FSH	KNN	0.99	0.99	1.0	0.97	0.99
Ferritin HbA1c FSH	KNN	0.99	0.99	1.0	0.97	0.99
Age UF number Ferritin HbA1c	LR	0.99	1	1.0	0.97	0.99
Age UF number Ferritin HbA1c	KNN	0.99	0.99	1.0	0.97	0.99
Age UF number Ferritin FSH	DT	0.99	0.99	1.0	0.97	0.99
Age UF number Ferritin FSH	RF	0.99	0.99	1.0	0.97	0.99
Age UF number Ferritin FSH	KNN	0.99	0.99	1.0	0.97	0.99
Age Ferritin HbA1c FSH	KNN	0.99	0.99	1.0	0.97	0.99
UF number Ferritin HbA1c FSH	KNN	0.99	0.99	1.0	0.97	0.99
Age UF number Ferritin HbA1c FSH	SVM	0.99	0.99	1.0	0.97	0.99

RF: Random Forest, KNN: K-nearest neighbors, FSH: Follicle stimulating hormone, UF number: Uterine fibroid number, SVM: Support Vector Machine, DT: Decision Tree, LR: Logistic Regression.

## Data Availability

The data produced and examined in the present study are available through the Istinye University Dataset Sharing Platform. De-identified clinical datasets may be accessed via the following link: https://dataset.istinye.edu.tr/dataset?did=83 (accessed on 1 February 2026). All records were anonymized in full compliance with applicable ethical standards. Data access is granted exclusively for research use within a controlled-access framework, in accordance with the platform’s established data-sharing and licensing policies. The shared dataset contains de-identified, processed variables suitable for analysis rather than raw clinical records to ensure compliance with ethical and data-protection standards.

## Supplementary Materials

The following supporting information can be downloaded at: https://www.mdpi.com/article/10.3390/medicina62030500/s1. Reference [31] is cited in Supplementary Materials.

## Abbreviations

Abbreviation	Definition
UF	Uterine Fibroid
UL	Uterine Leiomyoma
HbA1c	Glycated Hemoglobin
IGF-I	Insulin-like Growth Factor I
AMPK	AMP-Activated Protein Kinase
mTOR	Mammalian Target of Rapamycin
BMI	Body Mass Index
MetS	Metabolic Syndrome
HOMA-IR	Homeostasis Model Assessment of Insulin Resistance
FSH	Follicle-Stimulating Hormone
LH	Luteinizing Hormone
E2	Estradiol
PRL	Prolactin
AMH	Anti-Müllerian Hormone
SHBG	Sex Hormone-Binding Globulin
Hb	Hemoglobin
IDA	Iron Deficiency Anemia
CI	Confidence Interval
OR	Odds Ratio
AUC	Area Under the Curve
ROC	Receiver Operating Characteristic
ML	Machine Learning
AI	Artificial Intelligence
SVM	Support Vector Machine
DT	Decision Tree
RF	Random Forest
KNN	k-Nearest Neighbors
LR	Logistic Regression
TRIPOD-AI	Transparent Reporting of a multivariable prediction model for Individual Prognosis Or Diagnosis–Artificial Intelligence
PROBAST-AI	Prediction model Risk Of Bias ASsessment Tool–Artificial Intelligence

## References

[1] Ali M., Ciebiera M., Vafaei S., Alkhrait S., Chen H.-Y., Chiang Y.-F., Huang K.-C., Feduniw S., Hsia S.-M., Al-Hendy A. (2023). Progesterone Signaling and Uterine Fibroid Pathogenesis; Molecular Mechanisms and Potential Therapeutics. Cells.

[2] Flake G.P., Andersen J., Dixon D. (2003). Etiology and pathogenesis of uterine leiomyomas: A review. Environ. Health Perspect..

[3] Reis F.M., Bloise E., Ortiga-Carvalho T.M. (2016). Hormones and pathogenesis of uterine fibroids. Best. Pract. Res. Clin. Obstet. Gynaecol..

[4] Baird D.D., Travlos G., Wilson R., Dunson D.B., Hill M.C., D’Aloisio A.A., London S.J., Schectman J.M. (2009). Uterine leiomyomata in relation to insulin-like growth factor-I, insulin, and diabetes. Epidemiol. Camb. Mass..

[5] Uimari O., Auvinen J., Jokelainen J., Puukka K., Ruokonen A., Järvelin M.-R., Piltonen T., Keinänen-Kiukaanniemi S., Zondervan K., Järvelä I. (2016). Uterine fibroids and cardiovascular risk. Hum. Reprod. Oxf. Engl..

[6] Tak Y.J., Lee S.Y., Park S.K., Kim Y.J., Lee J.G., Jeong D.W., Kim S.C., Kim I.J., Yi Y.H. (2016). Association between uterine leiomyoma and metabolic syndrome in parous premenopausal women: A case-control study. Medicine.

[7] Takeda T., Sakata M., Isobe A., Miyake A., Nishimoto F., Ota Y., Kamiura S., Kimura T. (2008). Relationship between metabolic syndrome and uterine leiomyomas: A case-control study. Gynecol. Obstet. Investig..

[8] Hou Z.-M., Sun Q., Liu Y.-Z., Chen T.-F., Tang N. (2015). Effects of insulin resistance on myometrial growth. Int. J. Clin. Exp. Med..

[9] Mitro S.D., Waetjen L.E., Lee C., Wise L.A., Zaritsky E., Harlow S.D., El Khoudary S.R., Santoro N., Solomon D.H., Thurston R.C. (2025). Diabetes and Uterine Fibroid Diagnosis in Midlife: Study of Women’s Health Across the Nation (SWAN). J. Clin. Endocrinol. Metab..

[10] Li B., Takeda T., Tsuiji K., Kondo A., Kitamura M., Wong T.F., Yaegashi N. (2013). The antidiabetic drug metformin inhibits uterine leiomyoma cell proliferation via an AMP-activated protein kinase signaling pathway. Gynecol. Endocrinol. Off. J. Int. Soc. Gynecol. Endocrinol..

[11] Tseng C.-H. (2019). Metformin use is associated with a lower risk of uterine leiomyoma in female type 2 diabetes patients. Ther. Adv. Endocrinol. Metab..

[12] Sheu W.H.-H., Chen Y.-T., Lee W.-J., Wang C.-W., Lin L.-Y. (2003). A relationship between serum ferritin and the insulin resistance syndrome is present in non-diabetic women but not in non-diabetic men. Clin. Endocrinol..

[13] De Pergola G., Maldera S., Tartagni M., Pannacciulli N., Loverro G., Giorgino R. (2006). Inhibitory effect of obesity on gonadotropin, estradiol, and inhibin B levels in fertile women. Obes. Silver Spring Md..

[14] Rossetti A., Sizzi O., Soranna L., Cucinelli F., Mancuso S., Lanzone A. (2001). Long-term results of laparoscopic myomectomy: Recurrence rate in comparison with abdominal myomectomy. Hum. Reprod..

[15] La Verde M., Cobellis L., Torella M., Morlando M., Riemma G., Schiattarella A., Conte A., Ambrosio D., Colacurci N., De Franciscis P. (2022). Is Uterine Myomectomy a Real Contraindication to Vaginal Delivery? Results from a Prospective Study. J. Investig. Surg..

[16] Collins G.S., Moons K.G.M., Dhiman P., Riley R.D., Beam A.L., Van Calster B., Ghassemi M., Liu X., Reitsma J.B., van Smeden M. (2024). TRIPOD+AI statement: Updated guidance for reporting clinical prediction models that use regression or machine learning methods. BMJ.

[17] Moons K.G.M., Damen J.A.A., Kaul T., Hooft L., Andaur Navarro C., Dhiman P., Beam A.L., Van Calster B., Celi L.A., Denaxas S. (2025). PROBAST+AI: An updated quality, risk of bias, and applicability assessment tool for prediction models using regression or artificial intelligence methods. BMJ.

[18] Ahmad J., Rafat D. (2013). HbA1c and iron deficiency: A review. Diabetes Metab. Syndr..

[19] Silva J.F., Pimentel A.L., Camargo J.L. (2016). Effect of iron deficiency anaemia on HbA1c levels is dependent on the degree of anaemia. Clin. Biochem..

[20] Radin M.S. (2014). Pitfalls in hemoglobin A1c measurement: When results may be misleading. J. Gen. Intern. Med..

[21] AlQarni A.M., Alghamdi A.A., Aljubran H.J., Bamalan O.A., Abuzaid A.H., AlYahya M.A. (2023). The Effect of Iron Replacement Therapy on HbA1c Levels in Diabetic and Nondiabetic Patients: A Systematic Review and Meta-Analysis. J. Clin. Med..

[22] Kuang L., Li W., Xu G., You M., Wu W., Li C. (2021). Systematic review and meta-analysis: Influence of iron deficiency anemia on blood glycosylated hemoglobin in diabetic patients. Ann. Palliat. Med..

[23] Ma H., Lin H., Hu Y., Li X., He W., Jin X., Gao J., Zhao N., Pan B., Gao X. (2018). Serum ferritin levels are associated with insulin resistance in Chinese men and post-menopausal women: The Shanghai Changfeng study. Br. J. Nutr..

[24] Wrede C.E., Buettner R., Bollheimer L.C., Schölmerich J., Palitzsch K.-D., Hellerbrand C. (2006). Association between serum ferritin and the insulin resistance syndrome in a representative population. Eur. J. Endocrinol..

[25] Başar Gökcen B., Akdevelioğlu Y., Canan S., Bozkurt N. (2021). Evaluation of the relationship between serum ferritin and insulin resistance and visceral adiposity index (VAI) in women with polycystic ovary syndrome. Eat. Weight Disord..

[26] Luque-Ramírez M., Alvarez-Blasco F., Botella-Carretero J.I., Sanchón R., San Millán J.L., Escobar-Morreale H.F. (2007). Increased body iron stores of obese women with polycystic ovary syndrome are a consequence of insulin resistance and hyperinsulinism and are not a result of reduced menstrual losses. Diabetes Care.

[27] Saucedo R., Basurto L., Zarate A., Martínez C., Hernandez M., Galván R. (2007). Effect of estrogen therapy on insulin resistance and plasminogen activator inhibitor type 1 concentrations in postmenopausal women. Gynecol. Obstet. Investig..

[28] Saei Ghare Naz M., Farhadi-Azar M., Noroozzadeh M., Farahmand M., Ramezani Tehrani F. (2024). Follicle-Stimulating Hormone and Diabetes in Postmenopausal Women: A Systematic Review and Meta-Analysis. J. Clin. Endocrinol. Metab..

[29] Overgaard M., Glintborg D., Christesen H.T., Jensen T.K., Andersen M.S. (2020). Maternal prolactin is associated with glucose status and PCOS in pregnancy: Odense Child Cohort. Eur. J. Endocrinol..

[30] Sung J.-H., Kim K.-S., Han K., Park C.-Y. (2024). Association of Uterine Leiomyoma with Type 2 Diabetes Mellitus in Young Women: A Population-Based Cohort Study. Diabetes Metab. J..

[31] Debray T.P.A., Collins G.S., Riley R.D., Snell K.I.E., Van Calster B., Reitsma J.B., Moons K.G.M. (2023). Transparent reporting of multivariable prediction models developed or validated using clustered data: TRIPOD-Cluster checklist. BMJ.

